# Cardiac Ultrasound under Speckle Tracking Technology Based Analysis of Efficacy of Respiratory Rehabilitation on Chronic Obstructive Pulmonary Disease

**DOI:** 10.1155/2021/7569908

**Published:** 2021-08-12

**Authors:** Chuangen Ren, Wantian Liu

**Affiliations:** ^1^Department of Ultrasound, The First Affiliated Hospital of Wenzhou Medical University, Wenzhou 325000, Zhejiang, China; ^2^Department of Ultrasound Medicine, Wenzhou Traditional Chinese Medicine Affiliated to Zhejiang Chinese Medicine University, Wenzhou 325000, Zhejiang, China

## Abstract

The study shined spotlight on the effect of respiratory rehabilitation training on chronic obstructive pulmonary disease (COPD), which was evaluated using speckle ultrasound algorithm-based cardiac ultrasound. Then, 90 patients with stable COPD, who were admitted to the hospital from January 2018 to December 2019, were randomly rolled into three groups, namely, the fast inhalation and slow exhalation (A) group, abdominal breathing (B) group, and control (C) group. For group A, on the basis of the conventional treatment, the method of rapid inhalation and slow exhalation was adopted. The group B (*n* = 30) adopted the abdominal breathing method besides the conventional treatment. In addition, the group C (*n* = 30) received only conventional treatment. Finally, the efficacy and parameters of the three treatment methods were compared. The echocardiographic parameters and echocardiographic images were calculated and processed by the speckle tracking method. Three kinds of operators were used to track the myocardial spots successfully, and the corresponding points in the image were obtained and calculated. It was found that there was no significant difference in the degree of dyspnea, exercise endurance, lung function, respiratory muscle function, and quality of life (QOL) before treatment (*P* > 0.05). After treatment, in contrast with group C, the previously mentioned indicators in groups A and B were obviously better (*P* < 0.05). Further, both the echocardiographic images and echocardiographic parameters of groups A and B were obviously improved, and there was no obvious difference between groups A and B. Hence, some degree of respiratory rehabilitation was very effective in the diagnosis of patients with chronic pulmonary obstruction. In conclusion, the speckle tracking algorithm-based cardiac ultrasound improves the image quality. At the same time, respiratory rehabilitation training is effective on COPD and worthy of clinical promotion.

## 1. Introduction

The common symptoms of COPD are characterized by irreversible airflow limitation and difficulty in breathing, and patients often have more severe dyspnea at the onset. At the same time, as the disease progresses, the patient's airflow limitation aggravates, and this symptom is largely related to the inhalation of harmful gases and particles [1, 2]. As a modern disease, COPD has high fatality and disability rates.

With the development of modern medicine, ultrasound diagnosis is adopted in the detection of heart function in patients with COPD. At this stage, some speckles often appear in cardiac ultrasound images. For these speckles, there are two common points at this stage. One point is that it is a kind of noise that influences the image quality. The other point claims that it is a structural signal, which mainly reflects the information between the organ tissue and the imaging medium [3, 4]. Although the two viewpoints are quite different, their purpose is consistent, that is, for better diagnosis, research, and treatment of lesions. At the same time, due to the complexity of COPD itself, it often has complex effects on body functions, which not only acts on the respiratory system, but also has an impact on the structure of skeletal muscle as a result of inflammation in the body caused by COPD. Then, it causes dysfunctions in the respiratory muscles and further aggravates the patient's condition [5, 6]. The symptoms of weakened respiratory muscle function include decreased muscle function and decreased muscle endurance, which are often accompanied by dyspnea, increased carbon dioxide content in the blood, and decreased exercise capacity.

Further research on related phenomena shows that the increase in number of patients with reduced respiratory muscle function will greatly aggravate the consumption of medical resources, and the decline in respiratory muscle function also directly affects the survival rate of COPD patients [7, 8]. Therefore, it is necessary for COPD patients to exercise to improve their respiratory muscle function, and the way to realize it is to perform breathing training.

Therefore, the therapeutic effect of respiratory rehabilitation training on COPD patients was further studied utilizing speckle tracking algorithm-based ultrasound images.

## 2. Methods and Materials

### 2.1. Feature Point Operator Construction and Matching Pursuit

The speckles in cardiac ultrasound generally reflect the movement information of the epicardium and myocardium. At the same time, the speckles move with the movement of the myocardium. On this basis, the speckle information closely related to the movement of the myocardium is needed. In the study, the Forstner operator, SUSAN operator, and Harris operator were selected as the research subjects.

The Forstner operator is a well-known positioning operator in photogrammetry. It has the characteristics of fast speed and high accuracy. This operator takes the distance from the origin to the edge line of the inner edge of the window as the observed value, takes the gradient modulus square as the weight, and estimates the coordinates of the intersection point by the least square method. First, points with greater gray-scale differences from the surroundings were selected as feature points. The absolute values of the gray-scale differences in the four directions (up, down, left, and right) of the pixel point were calculated as follows:

(1) $$\begin{array}{c} \left\{\begin{array}{c} \text{d}f_{1}=\left|f_{a,b}-f_{a+1,b}\right|,\\ \text{d}f_{2}=\left|f_{a,b}-f_{a,b+1}\right|,\\ \text{d}f_{3}=\left|f_{a,b}-f_{a-1,b}\right|,\\ \text{d}f_{4}=\left|f_{a,b}-f_{a,b-1}\right|, \end{array}\right. \end{array}$$

where *f*_*a*,*b*_  is the gray value of (*a*, *b*), and *f*_*a*+1,*b*_, *f*_*a*,*b*+1_, *f*_*a*,*b*+1_, and *f*_*a*,*b*−1_ are the gray values of the four neighboring points of up, down, left, and right (*a*, *b*). Then, the mean value of these four absolute differences is calculated as *M*=mean{d*f*_1_, d*f*_2_, d*f*_3_, d*f*_4_}. For a given threshold *Y*, if *M* > *Y*, then (*a*, *b*) is the primary selected point.

In the 3 × 3 window with the primary selection point (*a*, *b*) as the center, the covariance matrix *T* and the interest value *I* are calculated according to the simplified Forstner operator, and the equations for *T* and *I* are as follows:

(2) $$\begin{array}{c} T=\left(\begin{array}{c} \sum g_{x}^{2}\sum g_{x}g_{y}\\ \sum g_{x}g_{y}\sum g_{y}^{2} \end{array}\right),\\ I=\frac{4\text{DeT}\left(T\right)}{{\left(\text{trT}\right)}^{2}}, \end{array}$$

where*g*_*x*_ and *g*_*y*_ are the Roberts gradients, Det(*T*) is the determinant of matrix *T*, trT is the trace of matrix *T* (the sum of diagonal elements), andtrT=∑*g*_*x*_^2^+∑*g*_*y*_^2^. *g*_*x*1_. The *g*_*x*1_ and *g*_*y*1_*g*_*y*2_ are as calculated as follows:

(3) $$\begin{array}{c} \left\{\begin{array}{c} g_{x1}=f\left(a,b\right)-f\left(a-1,b+1\right),\\ g_{x2}=f\left(a+1,b-1\right)-f\left(a,b\right),\\ g_{y1}=f\left(a,b\right)-f\left(a+1,b+1\right),\\ g_{y2}=f\left(a-1,b-1\right)-f\left(a,b\right). \end{array}\right. \end{array}$$

The SUSAN operator adopts the USAN corner detection, that is, the pixel to be detected in the center of the circular window template, is called core point. The SUSAN template slides over the image, and the gray value of the image pixel in the template is compared with the gray value of the core point at each position:

(4) $$\begin{array}{c} C\left(x,x_{0}\right)=\left\{\begin{array}{cc} 1, & \left|Z\left(x\right)-Z\left(x_{0}\right)\right|\leq t,\\ 0, & \left|Z\left(x\right)-Z\left(x_{0}\right)\right|\geq t, \end{array}\right. \end{array}$$

where *x*_0_ is the position of the core point of the image, *x* is the position of other points in the template, *Z*_0_ is the gray value function, *t* is the threshold to restore to the difference value, and *c*(*x*, *x*_0_) is the comparison result. The brightness difference between points in the template and the core point is calculated, and the sum is as follows:

(5) $$\begin{array}{c} n\left(x_{0}\right)=\sum c\left(x,x_{0}\right). \end{array}$$

Then, *n* is compared with a given threshold, and SUSAN sets *g* as half of the size of *n*_max_. Finally, the local maximum value of the corner response is obtained, and the corresponding pixel is marked as a corner point.

Inspired by the autocorrelation function in the signal processing, Harris operator gives the matrix *N* related to the autocorrelation function. The eigenvalue of the *N* matrix is the first-order curvature of the autocorrelation. If the two curvature values are both high, the point is considered to be a feature point, and the expression of the Harris operator is as follows:

(6) $$\begin{array}{c} M=G\left(\tilde{s}\right)⊗\left[\begin{array}{cc} g_{u} & g_{u}g_{v}\\ g_{u}g_{v} & g_{v} \end{array}\right], \end{array}$$

where *q*=Det(*T*) − *k*Trac^2^(*T*), *k* = 0.04, *g*_*u*_ is the gradient in the direction of *x*, *g*_*v*_ is the gradient in the direction of *y*, $G\left(\tilde{s}\right)$ is the Gaussian template, ⊗ is the convolution operation, *q* is the interest value of each point, DeT is the determinant of the matrix, Trac is the trace of the matrix, and *k* is the threshold.

### 2.2. Research Subjects and Groups

Ninety COPD patients admitted to our hospital from January 2018 to December 2019 were selected. All patients admitted met the following criteria: (i) patients had clinically confirmed COPD; (ii) they were older than 50 years and younger than 75 years; (iii) they had normal liver and kidney functions. Patients with the following conditions were excluded: (i) those who had type I or II respiratory failure; (ii) those who had pleural disease or thoracic deformity, bone and joint disease, and neuromuscular junction disease; (iii) those who had received other treatments including traditional Chinese medicine; (iv) those who refused to receive rehabilitation training and had poor compliance. All patients participating in the experiment signed informed consent forms, and this study got permission from the hospital ethics committee.

Ninety patients were randomly divided into three groups, each with 30 cases, namely, the fast inhalation and slow exhalation (A) group, abdominal breathing (B) group, and control (C) group. For group A, on the basis of the conventional treatment, the method of rapid inhalation and slow exhalation was adopted. The group B (*n* = 30) adopted the abdominal breathing method besides the conventional treatment. In addition, the group C (*n* = 30) received only conventional treatment.

### 2.3. Respiratory Rehabilitation Training Methods

The three groups were all treated with conventional medical treatment, which lasted for eight weeks. On this basis, the group A accepted fast inhalation and slow-exhalation training, and the group B accepted abdominal breathing training. Fast inhalation and slow exhalation training method: the patient was asked breathe quickly through the nose, breathe as much as possible until the lungs were saturated, and the saturated state should be maintained for a short time. Then, the patient breathed out slowly. The exhalation process was also completed through the nose, and the whole breathing was about five seconds, and the time ratio of inhalation and exhalation was 1 : 3 or 1 : 4. Abdominal breathing training method was as the patient's left hand was placed on the chest and the right hand on the upper abdomen. The patient should keep the abdomen swelling when inhaling and make the abdomen actively collapse when exhaling and place the right hand on the back when exhaling, to give a certain pressure in the direction so that the diaphragm can effectively recover, while the left hand remains motionless during the entire breathing process.

### 2.4. Related Testing Indicators

The basic examinations for all selected patients included lung ventilation function, respiratory muscle function, exercise endurance test, QOL score, and dyspnea score. Among them, the QOL score was measured using SGRQ, the score was within 0–100 points, and the score was negatively correlated with the patient's health status.

The functional dyspnea score was based on the British medical research council (MRC) dyspnea scale. Level 0 means dyspnea during hard exercise; level 1 means shortness of breath when people walk on flat ground or on small slopes; level 2 is shortness of breath when people walk on flat ground; therefore, they are slower than others of the same age and need to stop and rest; level 3 means walking on flat ground for about 100 meters or a few minutes and needs to stop and breathe; level 4 means being unable to leave home due to severe breathing difficulties or having difficulties putting on or taking off clothes.

### 2.5. Statistical Analysis

The SPSS 22.0 was adopted for statistical analysis, and the mean ± standard deviation was adopted to express the counting results. The comparison between the two groups was realized by *t*-test, and *P* < 0.05 was statistically significant.

## 3. Results

### 3.1. Feature Point Extraction and Matching of Speckle Tracking Algorithm

**Figure 1:**
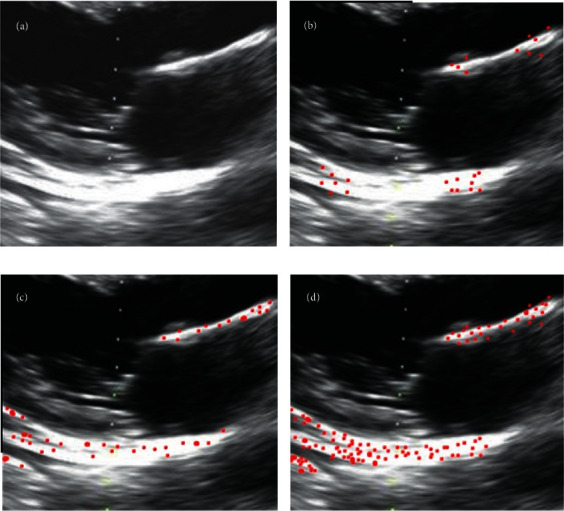
Feature point extraction effect.

The extraction results of the feature points of the echocardiogram by the Forstner operator, SUSAN operator, and Harris operator were compared, and the results are shown in Figure 1.  Figure 1(a) is the first image of the short-axis motion of the left ventricle during diastole in echocardiography, Figure 1(b) is the image processed by the Forstner operator, Figure 1(c) is the image processed by the SUSAN operator, and Figure 1(d) is the image processed by the Harris operator. It was evident that the SUSAN operator and Harris operator were obviously better than the Forstner operator in extracting features at the corners of the contour. At the same time, the running time of the Forstner operator algorithm was 2.054 s, that of the SUSAN operator algorithm was 2.291 s, and that of the Harris operator was 3.249 s (*P* < 0.05).

### 3.2. General Information

**Figure 2:**
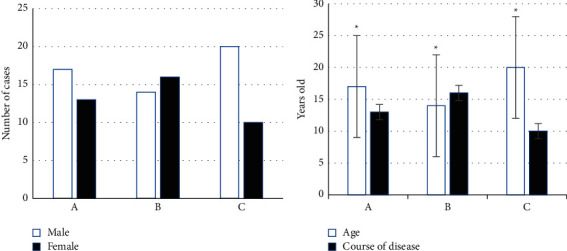
Comparison of general patient data. (a) Gender; (b) age and course of disease (^*∗*^ means that the difference was statistically significant *P* < 0.05).

This study included 90 patients in the experiment, including 30 in groups A, B, and C, respectively. Among them, there were 17 males and 13 females in group A. They were 65.17 ± 5.78 years old in average, and the average course of disease was 8.17 ± 1.24 years; in group B, there were 14 males and 16 females, and they were 66.45 ± 7.22 years old in average, with an average course of 8.73 ± 1.34 years; group C included 20 males and 10 females, and they were 66.14 ± 6.17 years old in average, and the average course of disease was 8.01 ± 1.13 years. During the experiment, there were no patients who were omitted to follow-up, and the loss-to-follow-up rate was 0%. Then, the previously mentioned information was compared (*P* < 0.05) (Figure 2).

### 3.3. The Difficulty to Learn Respiratory Rehabilitation Training

**Figure 3:**
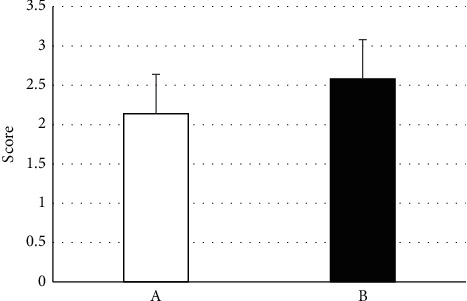
Learning difficulty of different breathing rehabilitation training.

Groups A and B undergoing breathing rehabilitation training were evaluated for the difficulty of learning. The lower the score, the easier it was to master, and vice versa. The results are shown in Figure 3. The score of group A was 2.14 ± 0.57, and that of group B was 2.58 ± 0.95 (*P* > 0.05).

### 3.4. Changes in Exercise Endurance and Dyspnea

**Figure 4:**
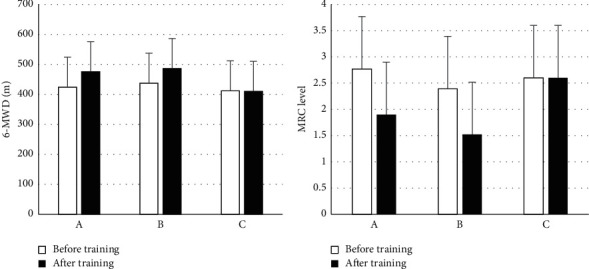
Changes in exercise endurance and dyspnea. (a) Exercise endurance; (b) difficulty breathing.

Figure 4 shows the changes in exercise endurance and dyspnea before and after breathing rehabilitation training, and there was no obvious difference in exercise endurance and dyspnea before the three groups of patients (*P* > 0.05). After training, in contrast with C, patients in groups A and B had a significant increase in exercise endurance and a significant improvement in dyspnea (*P* < 0.05), and the difference between groups A and B was not obvious (*P* > 0.05).

### 3.5. Changes in Lung Function

**Figure 5:**
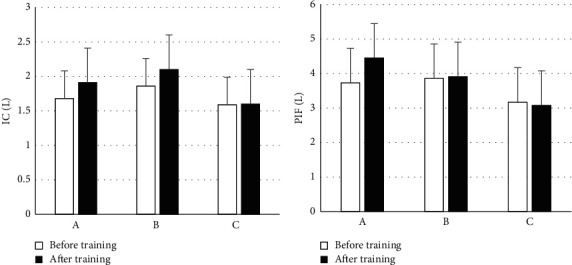
Changes in lung function. (a) Inspiratory volume (IC); (b) peak inspiratory flow (PIF) rate.

The changes in lung function before and after respiratory rehabilitation training are shown in Figure 5. There was no obvious difference between the deep inspiratory volume (IC) and peak inspiratory flow rate (PIF) of patients in groups A, B, and C before training (*P* > 0.05). After training, in contrast with group C, the IC and PIF of groups A and B were obviously increased (*P* < 0.05), and there was no obvious difference between groups A and B (*P* > 0.05).

### 3.6. Changes in Cardiac Function

**Figure 6:**
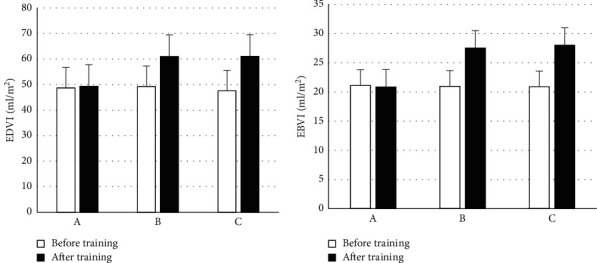
Changes in cardiac function. (a) EDVI; (b) ESVI.

This part of the detection was carried out using the spot tracking algorithm, and the changes in the heart function before and after the respiratory rehabilitation training are shown in Figure 6. Before training, the difference between left ventricular EDVI and left ventricular ESVI was not obvious (*P* > 0.05) in groups A, B, and C. After training, in contrast with group C, the EDVI and ESVI of groups A and B were obviously reduced (*P* < 0.05), and the difference between the groups A and B was not obvious (*P* > 0.05).

### 3.7. Changes in Respiratory Muscle Function

**Figure 7:**
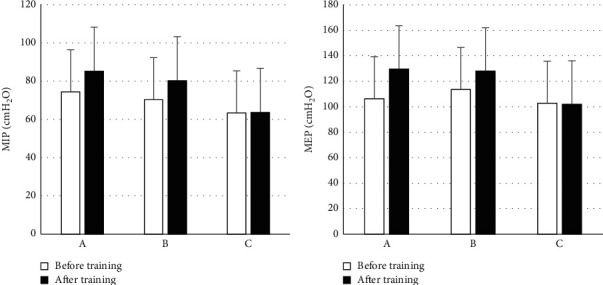
Changes in respiratory muscle function. (a) MIP; (b) MEP.

The changes in respiratory muscle function before and after respiratory rehabilitation training are shown in Figure 7. There was no obvious difference between pretraining MIP and MEP in groups A, B, and C (*P* > 0.05). After training, in contrast with group C, the MIP and MEP of groups A and B were obviously increased (*P* < 0.05), and there was no obvious difference between groups A and B (*P* > 0.05).H_2_O

### 3.8. Changes in the QOL

**Figure 8:**
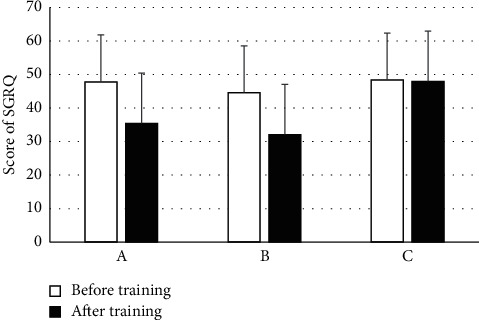
Changes in QOL.

The changes in the QOL before and after respiratory rehabilitation training are indicated in Figure 8. The overall QOL in groups A, B, and C before training was low (*P* > 0.05). After training, in contrast with group C, the QOL of groups A and B had a significant increase (*P* < 0.05), and there was no obvious difference between groups A and B (*P* > 0.05).

## 4. Discussion

At this stage, the main treatment methods for patients in the stable phase of COPD are drug therapy and nondrug therapy, and respiratory rehabilitation therapy is not the mainstream treatment method [9]. COPD patients often have problems not only with lung function, but also with heart function. In the study, speckle tracking algorithm was adopted to analyze cardiac ultrasound. It was found that the point of motion between adjacent frames was small, and it was limited to calculate the elastic strain parameter rotation angle of the local area according to this. However, it was sensible to calculate the movement displacement length by combining the movement displacement vector of each point in the movement process. Movement restriction is one of the main symptoms that can be observed in patients with COPD. Due to functional impairment of the respiratory muscles, the patient often suffers a significant increase in load at the same time, which in turn causes that the patient's ventilation volume is unable to meet the increased demand for ventilation during exercise [10]. Berry et al. showed that systematic breathing training can obviously improve the patient's exercise endurance. In this study, the 6-MWD of groups A and B were obviously increased compared with before training, indicating that the two training methods can also improve the exercise endurance of patients with COPD [11]. It was confirmed in the study that both breathing training methods can increase the patient's IC value, suggesting that the increase in IC may be an important reason for the improvement of patients' exercise endurance. In addition, exercise endurance is not only related to the patient's ventilation ability, but also related to the function of the limbs and respiratory muscles. Langer et al. found that MIP was an important factor affecting exercise endurance in patients with severe COPD, indicating that respiratory muscle function was related to exercise endurance. In this study, the respiratory muscle strength of the two groups of patients was obviously improved compared with before training [12].

Due to the particularity of the disease itself, COPD often manifests as a complex respiratory system disease, accompanied by chronic disease of multiple organs throughout the body, which affects a variety of extrapulmonary tissues including skeletal muscle [13]. Due to the appearance of inflammation, the system has an impact on the structure of organs including skeletal muscles, which further leads to the dysfunction of the body including respiratory muscles [14]. Respiratory muscle dysfunction is often caused by a combination of many factors and, to a large extent, is closely related to the clinical manifestations of COPD patients and the severity of the disease [15]. Breathing training can improve the breathing of patients. The study of Kerti et al. showed that, after breathing muscle training in COPD patients, MIP increased obviously before training [16]. In this study, the MIP and MEP of patients in groups A and B were obviously increased than before training, suggesting that both training methods can improve the respiratory muscle function of COPD patients.

## 5. Conclusion

In this research, the three operators of the speckle tracking algorithm were studied and verified accordingly. Then, the therapeutic effect of respiratory rehabilitation training on COPD patients was further explored, and it was found that, in contrast with the control group, the degree of dyspnea, exercise endurance, lung function, respiratory muscle function, and life quality were obviously improved (*P* < 0.05). In the study, MIP and MEP in group A and group B were significantly higher than those before training, indicating that both the two training methods can improve the respiratory muscle function of COPD patients. However, the research on the algorithm in this study is limited to cardiac ultrasound images. Whether the speckle tracking algorithm is useful for other ultrasound images and needs to be further explored. At the same time, the experimental research samples are small and the types of patients are not subdivided, which should be improved in the future.

## Data Availability

The data used to support the findings of this study are available from the corresponding author upon request.
